# Bis(2,9-dimethyl-1,10-phenanthrolin-1-ium) 2,5-di­carb­oxy­benzene-1,4-di­carb­oxyl­ate–2,9-dimethyl-1,10-phenanthroline–benzene-1,2,4,5-tetra­carb­oxy­lic acid (1/2/1)

**DOI:** 10.1107/S1600536813022691

**Published:** 2013-08-17

**Authors:** Hadi D. Arman, Trupta Kaulgud, Edward R. T. Tiekink

**Affiliations:** a Department of Chemistry, The University of Texas at San Antonio, One UTSA Circle, San Antonio, Texas 78249-0698, USA; b Department of Chemistry, University of Malaya, 50603 Kuala Lumpur, Malaysia

## Abstract

The asymmetric unit of the title co-crystal, 2C_14_H_13_N_2_^+^·C_10_H_4_O_8_^2−^·2C_14_H_12_N_2_·C_10_H_6_O_8_, comprises a 2,9-dimethyl-1,10-phenanthrolin-1-ium cation (Me_2_PhenH^+^) and a 2,9-dimethyl-1,10-phenanthroline mol­ecule (Me_2_Phen), each in a general position, and half each of a 2,5-di­carb­oxy­benzene-1,4-di­carboxyl­ate dianion (*L*H_2_^2−^) and a benzene-1,2,4,5-tetra­carb­oxy­lic acid mol­ecule (*L*H_4_), each being disposed about a centre of inversion. Small twists are evident in the dianion [the C—C—C—O torsion angles are 168.41 (18) and 16.2 (3)°], whereas a major twist is found for one carb­oxy­lic acid group in the neutral mol­ecule [C—C—C—O = 66.3 (2) and 18.2 (3)°]. The most prominent feature of the crystal packing is the formation of linear supra­molecular chains along [001] mediated by charge-assisted O—H⋯O^−^ hydrogen bonding between alternating *L*H_4_ and *L*H_2_^2−^. These are connected to the Me_2_PhenH^+^ and Me_2_Phen species by N—H⋯O and O—H⋯N hydrogen bonds, respectively. A three-dimensional architecture is formed by C—H⋯O and π–π inter­actions [inter-centroid distance = 3.5337 (17) Å].

## Related literature

For salt formation with benzene-1,2,4,5-tetra­carb­oxy­lic acid, see: Arman & Tiekink (2013 ▶). For a co-crystal involving 2,9-dimethyl-1,10-phenanthroline, see: Arman *et al.* (2010 ▶). For the structure of a 2,9-dimethyl-1,10-phenanthrolin-1-ium carb­oxyl­ate salt, see: Derikvand & Olmstead (2011 ▶).
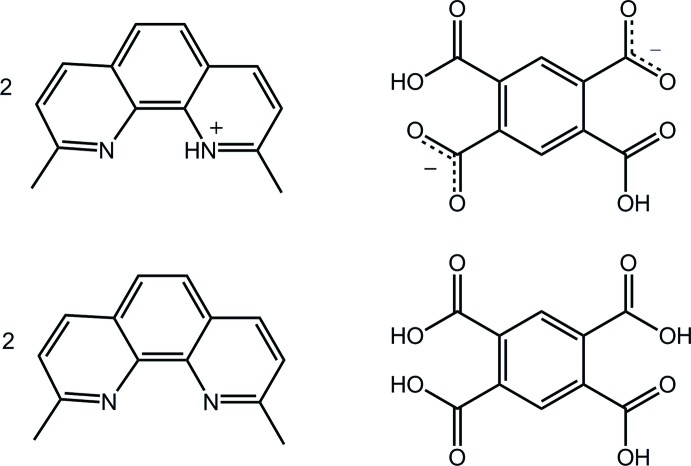


## Experimental

### 

#### Crystal data

2C_14_H_13_N_2_^+^·C_10_H_4_O_8_^2−^·2C_14_H_12_N_2_·C_10_H_6_O_8_
*M* *_r_* = 1341.32
Monoclinic, 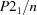

*a* = 11.798 (4) Å
*b* = 13.893 (4) Å
*c* = 19.163 (6) Å
β = 92.216 (5)°
*V* = 3138.8 (16) Å^3^
*Z* = 2
Mo *K*α radiation
μ = 0.10 mm^−1^
*T* = 98 K
0.48 × 0.37 × 0.09 mm

#### Data collection

Rigaku AFC12/SATURN724 diffractometer
Absorption correction: multi-scan (*ABSCOR*; Higashi, 1995 ▶) *T*_min_ = 0.723, *T*_max_ = 1.000
22006 measured reflections
7181 independent reflections
6007 reflections with *I* > 2σ(*I*)
*R*_int_ = 0.056

#### Refinement

*R*[*F* ^2^ > 2σ(*F* ^2^)] = 0.062
*wR*(*F* ^2^) = 0.148
*S* = 1.11
7181 reflections
467 parameters
4 restraints
H atoms treated by a mixture of independent and constrained refinement
Δρ_max_ = 0.29 e Å^−3^
Δρ_min_ = −0.25 e Å^−3^

### 

Data collection: *CrystalClear* (Molecular Structure Corporation & Rigaku, 2005 ▶); cell refinement: *CrystalClear*; data reduction: *CrystalClear*; program(s) used to solve structure: *SHELXS97* (Sheldrick, 2008 ▶); program(s) used to refine structure: *SHELXL97* (Sheldrick, 2008 ▶); molecular graphics: *ORTEPII* (Johnson, 1976 ▶) and *DIAMOND* (Brandenburg, 2006 ▶); software used to prepare material for publication: *publCIF* (Westrip, 2010 ▶).

## Supplementary Material

Crystal structure: contains datablock(s) global, I. DOI: 10.1107/S1600536813022691/xu5731sup1.cif

Structure factors: contains datablock(s) I. DOI: 10.1107/S1600536813022691/xu5731Isup2.hkl

Supplementary material file. DOI: 10.1107/S1600536813022691/xu5731Isup3.cml

Additional supplementary materials:  crystallographic information; 3D view; checkCIF report

## Figures and Tables

**Table 1:** Hydrogen-bond geometry (Å, °)

*D*—H⋯*A*	*D*—H	H⋯*A*	*D*⋯*A*	*D*—H⋯*A*
O4—H1o⋯O1	0.85 (2)	1.55 (2)	2.403 (2)	176 (3)
O6—H2o⋯O2^i^	0.85 (1)	1.74 (1)	2.577 (2)	168 (2)
O8—H3o⋯N4^ii^	0.85 (2)	1.79 (2)	2.636 (2)	173 (2)
N1—H1n⋯O3^iii^	0.89 (2)	2.41 (2)	3.257 (2)	161 (2)
N1—H1n⋯O4^iii^	0.89 (2)	2.35 (2)	2.957 (2)	126 (2)
C13—H13⋯O7^iv^	0.95	2.28	3.225 (3)	171
C28—H28⋯O5^v^	0.95	2.40	3.320 (3)	162

Symmetry codes: (i) 
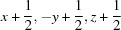
; (ii) 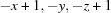; (iii) 
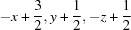
; (iv) 
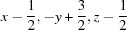
; (v) 
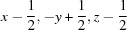
.

**Fig. 1.**
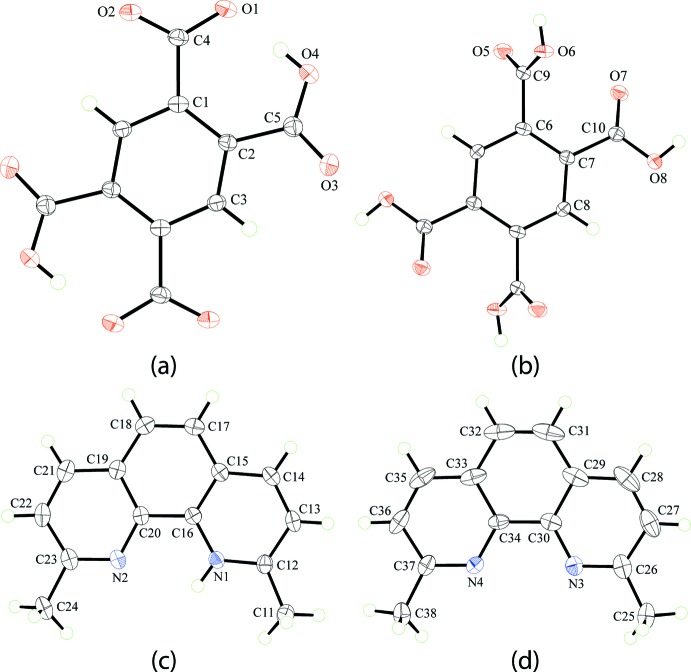
Molecular structures of the components of (I), showing atom-labelling scheme and displacement ellipsoids at the 50% probability level: (a) LH22-, (b) LH4, (c) Me2PhenH+ and (d) Me2Phen.

**Fig. 2.**
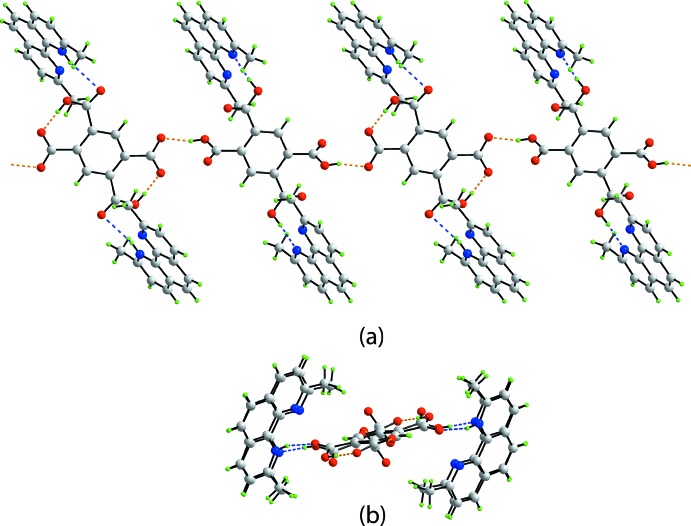
Views (a) side-on and (b) end-on of the supramolecular chain in (I). The O—H···O (orange), O—H···N (blue) and N—H···O (blue) hydrogen bonds are shown as dashed lines.

**Fig. 3.**
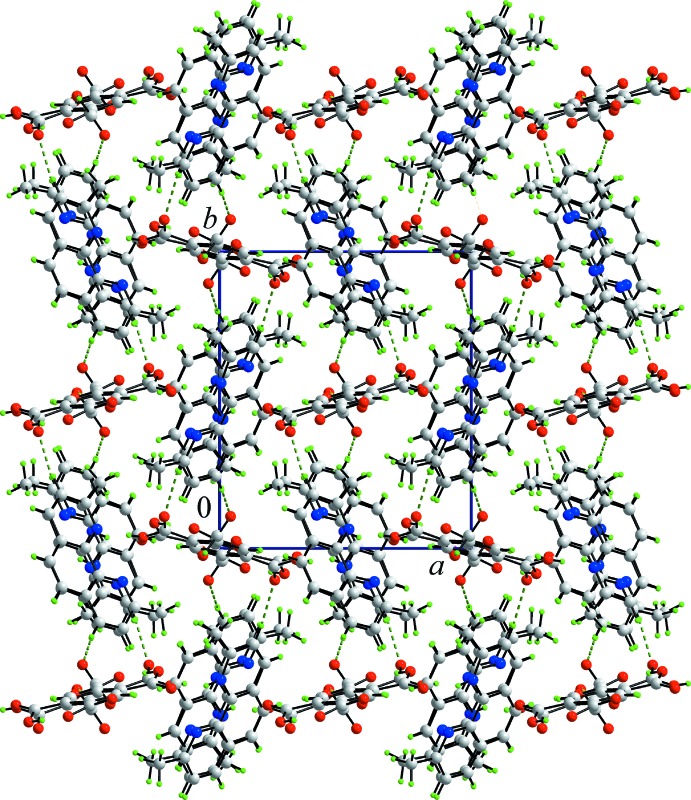
Unit-cell contents in (I) viewed in projection down the c axis. The C—H···O interactions are shown green dashed lines.

## supplementary crystallographic information

### 1. Comment

In continuation of on-going structural studies of salts/co-crystals formed between carboxylic acids and various pyridyl derivatives (Arman *et al.*, 2010; Arman & Tiekink, 2013), the title salt co-crystal, (I), was isolated from the 2:3 co-crystallization of benzene-1,2,4,5-tetracarboxylic acid (*L*H_4_) and 2,9-dimethyl-1,10-phenanthroline (Me_2_Phen).

The asymmetric unit of (I) comprises a centrosymmetric, doubly deprotonated *L*H_2_^2-^ dianion, a centrosymmetric neutral *L*H_4_ molecule, a protonated Me_2_PhenH^+^ cation and a neutral Me_2_Phen molecule, Fig. 1, and is formulated as a combination of a 2:1 Me_2_Phen^+^:*L*H_2_^2-^ salt combined with a 2:1 Me_2_Phen:HL_4_ co-crystal. A salt formed between Me_2_PhenH^+^ and a hydrogen(*S*,*S*)-tartrate has been reported (Derikvand & Olmstead, 2011).

Small twists are evident in the *L*H_2_^2-^ dianion as seen in the C2—C1—C4—O2 and C1—C2—C5—O4 torsion angles of 168.41 (18) and 16.2 (3)°, respectively. This arrangement is stabilized by intramolecular O—H···O hydrogen bonds, Table 1. By contrast, a considerable twist is evident in *L*H_4_ with the C7—C6—C9—O6 and C6—C7—C10—O7 torsion angles being 66.3 (2) and 18.2 (3)°, respectively. Such variations in conformation have been discussed in some detail (Arman & Tiekink, 2013). The Me_2_Phen molecule and Me_2_PhenH^+^ cation are each planar with the r.m.s. deviation for the 16 non-hydrogen atoms being 0.037 and 0.036 Å, respectively.

The prominent feature of the crystal packing is the formation of linear supramolecular chains along [0 0 1] comprising alternating *L*H_4_ and *L*H_2_^2-^ species connected *via* charge-assisted O6—H···O2 hydrogen bonding, Table 1. The hydroxyl-O8 forms an O—H···N4 hydrogen bond with the neutral Me_2_Phen molecules, one to either side of the carboxylic acid/carboxylate chain. The O3,O4 carboxylic acid residue accepts hydrogen bonds from the N1—H1n atom of the Me_2_PhenH^+^ cation, again, from symmetry, one to either side, leading to the supramolecular chain shown in Fig. 2a; an end-on view is shown in Fig. 2b. The Me_2_Phen and Me_2_PhenH^+^ cations inter-digitate along the *c* axis and are connected by π—π [*Cg*(C15–C20)···*Cg*(C29—C34)^i^ = 3.5337 (17) Å for *i*: *x*, 1 + *y*, *z*] interactions between Me_2_PhenH^+^ and Me_2_Phen. Additional contacts are of the type C—H···O, Table 1, as illustrated in the crystal packing diagram, Fig. 3.

### 2. Experimental

Crystals of (I) were obtained by the co-crystallization of benzene-1,2,4,5-tetracarboxylic acid (Sigma-Aldrich), 0.06 mmol) and 2,9-dimethylphenanthroline (ACROS, 0.09 mmol) in ethanol solution. Crystals were obtained by slow evaporation.

### 3. Refinement

C-bound H-atoms were placed in calculated positions (C—H = 0.95–0.98 Å) and were included in the refinement in the riding model approximation with *U*_iso_(H) set to 1.2–1.5*U*_eq_(C). The O-and N-bound H-atoms were located in a difference Fourier map and were refined with a distance restraints of O—H = 0.84±0.01 Å and N—H = 0.88±0.01 Å, and with *U*_iso_(H) = 1.2*U*_eq_(N) and 1.5*U*_eq_(O). Owing to being affected by the beam-stop, three reflections, *i.e*. (0 0 1), (1 0 1) and (-6 0 2), were omitted from the final cycles of refinement.

### Crystal data

**Table:**

2C_14_H_13_N_2_^+^·C_10_H_4_O_8_^2^^−^·2C_14_H_12_N_2_·C_10_H_6_O_8_	*F*(000) = 1400
*M**_r_* = 1341.32	*D*_x_ = 1.419 Mg m^−^^3^
Monoclinic, *P*2_1_/*n*	Mo *K*α radiation, λ = 0.71069 Å
Hall symbol: -P 2yn	Cell parameters from 12762 reflections
*a* = 11.798 (4) Å	θ = 2.0–40.7°
*b* = 13.893 (4) Å	µ = 0.10 mm^−^^1^
*c* = 19.163 (6) Å	*T* = 98 K
β = 92.216 (5)°	Prism, colourless
*V* = 3138.8 (16) Å^3^	0.48 × 0.37 × 0.09 mm
*Z* = 2	

### Data collection

**Table:**

Rigaku AFC12K/SATURN724 diffractometer	7181 independent reflections
Radiation source: fine-focus sealed tube	6007 reflections with *I* > 2σ(*I*)
Graphite monochromator	*R*_int_ = 0.056
ω scans	θ_max_ = 27.5°, θ_min_ = 2.1°
Absorption correction: multi-scan (*ABSCOR*; Higashi, 1995)	*h* = −15→15
*T*_min_ = 0.723, *T*_max_ = 1.000	*k* = −18→18
22006 measured reflections	*l* = −18→24

### Refinement

**Table:**

Refinement on *F*^2^	Primary atom site location: structure-invariant direct methods
Least-squares matrix: full	Secondary atom site location: difference Fourier map
*R*[*F*^2^ > 2σ(*F*^2^)] = 0.062	Hydrogen site location: inferred from neighbouring sites
*wR*(*F*^2^) = 0.148	H atoms treated by a mixture of independent and constrained refinement
*S* = 1.11	*w* = 1/[σ^2^(*F*_o_^2^) + (0.0555*P*)^2^ + 1.3348*P*] where *P* = (*F*_o_^2^ + 2*F*_c_^2^)/3
7181 reflections	(Δ/σ)_max_ < 0.001
467 parameters	Δρ_max_ = 0.29 e Å^−^^3^
4 restraints	Δρ_min_ = −0.25 e Å^−^^3^

### Special details

**Table:**

Geometry. All s.u.'s (except the s.u. in the dihedral angle between two l.s. planes) are estimated using the full covariance matrix. The cell s.u.'s are taken into account individually in the estimation of s.u.'s in distances, angles and torsion angles; correlations between s.u.'s in cell parameters are only used when they are defined by crystal symmetry. An approximate (isotropic) treatment of cell s.u.'s is used for estimating s.u.'s involving l.s. planes.
Refinement. Refinement of *F*^2^ against ALL reflections. The weighted *R*-factor *wR* and goodness of fit *S* are based on *F*^2^, conventional *R*-factors *R* are based on *F*, with *F* set to zero for negative *F*^2^. The threshold expression of *F*^2^ > 2σ(*F*^2^) is used only for calculating *R*-factors(gt) *etc*. and is not relevant to the choice of reflections for refinement. *R*-factors based on *F*^2^ are statistically about twice as large as those based on *F*, and *R*- factors based on ALL data will be even larger.

### Fractional atomic coordinates and isotropic or equivalent isotropic displacement parameters (Å^2^)

**Table:**

	*x*	*y*	*z*	*U*_iso_*/*U*_eq_	
O1	0.60101 (12)	0.56642 (11)	0.32029 (7)	0.0298 (3)	
O2	0.42698 (12)	0.50799 (11)	0.31498 (7)	0.0271 (3)	
O3	0.80815 (11)	0.52832 (11)	0.50668 (7)	0.0276 (3)	
O4	0.75764 (12)	0.58534 (11)	0.40310 (7)	0.0275 (3)	
H1O	0.7039 (17)	0.5802 (19)	0.3723 (11)	0.041*	
O5	1.04388 (12)	0.10773 (11)	0.67551 (7)	0.0279 (3)	
O6	0.93416 (12)	−0.02423 (10)	0.68129 (6)	0.0225 (3)	
H2O	0.941 (2)	−0.0150 (17)	0.7251 (5)	0.034*	
O7	0.78053 (11)	0.10681 (9)	0.60744 (6)	0.0217 (3)	
O8	0.69585 (11)	0.03734 (10)	0.51309 (7)	0.0225 (3)	
H3O	0.6399 (14)	0.0441 (18)	0.5394 (10)	0.034*	
N1	0.49493 (13)	1.05719 (11)	0.10595 (8)	0.0216 (3)	
H1N	0.5525 (13)	1.0368 (16)	0.0816 (10)	0.026*	
N2	0.59146 (14)	0.87982 (12)	0.11607 (8)	0.0242 (4)	
N3	0.38546 (15)	0.11239 (12)	0.39446 (8)	0.0251 (4)	
N4	0.48691 (13)	−0.06451 (12)	0.41450 (8)	0.0212 (3)	
C1	0.51592 (16)	0.51633 (13)	0.42769 (9)	0.0183 (4)	
C2	0.61045 (15)	0.52086 (13)	0.47597 (9)	0.0174 (3)	
C3	0.59041 (15)	0.50468 (13)	0.54626 (9)	0.0185 (4)	
H3	0.6533	0.5082	0.5787	0.022*	
C4	0.51495 (16)	0.53048 (14)	0.34896 (9)	0.0206 (4)	
C5	0.73412 (16)	0.54460 (14)	0.46160 (10)	0.0215 (4)	
C6	0.99235 (15)	0.02306 (12)	0.57069 (9)	0.0172 (3)	
C7	0.89506 (15)	0.02660 (12)	0.52636 (9)	0.0170 (3)	
C8	0.90372 (15)	0.00341 (13)	0.45593 (9)	0.0177 (4)	
H8	0.8380	0.0057	0.4257	0.021*	
C9	0.99120 (15)	0.04220 (13)	0.64807 (9)	0.0191 (4)	
C10	0.78392 (15)	0.06034 (13)	0.55334 (9)	0.0182 (4)	
C11	0.51318 (18)	1.21161 (15)	0.04831 (11)	0.0287 (4)	
H11A	0.5737	1.2474	0.0733	0.043*	
H11B	0.4577	1.2569	0.0277	0.043*	
H11C	0.5457	1.1731	0.0112	0.043*	
C12	0.45601 (16)	1.14676 (14)	0.09806 (10)	0.0234 (4)	
C13	0.36476 (17)	1.17594 (14)	0.13837 (10)	0.0261 (4)	
H13	0.3340	1.2388	0.1328	0.031*	
C14	0.32018 (17)	1.11385 (15)	0.18567 (10)	0.0258 (4)	
H14	0.2590	1.1343	0.2128	0.031*	
C15	0.36416 (16)	1.01985 (14)	0.19451 (10)	0.0220 (4)	
C16	0.45351 (16)	0.99289 (13)	0.15246 (10)	0.0206 (4)	
C17	0.32532 (16)	0.95298 (15)	0.24508 (10)	0.0245 (4)	
H17	0.2658	0.9708	0.2744	0.029*	
C18	0.37276 (17)	0.86434 (15)	0.25149 (10)	0.0253 (4)	
H18	0.3459	0.8209	0.2854	0.030*	
C19	0.46257 (16)	0.83521 (13)	0.20811 (10)	0.0225 (4)	
C20	0.50465 (16)	0.89957 (13)	0.15806 (10)	0.0214 (4)	
C21	0.51552 (18)	0.74414 (15)	0.21224 (11)	0.0284 (4)	
H21	0.4910	0.6975	0.2446	0.034*	
C22	0.60207 (18)	0.72363 (15)	0.16957 (11)	0.0301 (5)	
H22	0.6371	0.6621	0.1717	0.036*	
C23	0.64006 (17)	0.79384 (15)	0.12197 (11)	0.0271 (4)	
C24	0.73845 (19)	0.77429 (16)	0.07645 (12)	0.0353 (5)	
H24A	0.7311	0.8138	0.0342	0.053*	
H24B	0.7389	0.7061	0.0635	0.053*	
H24C	0.8095	0.7903	0.1020	0.053*	
C25	0.2311 (2)	0.21602 (17)	0.42569 (13)	0.0394 (6)	
H25A	0.1657	0.1888	0.3995	0.059*	
H25B	0.2202	0.2855	0.4314	0.059*	
H25C	0.2385	0.1854	0.4717	0.059*	
C26	0.3371 (2)	0.19826 (15)	0.38637 (11)	0.0310 (5)	
C27	0.3831 (2)	0.27008 (16)	0.34338 (12)	0.0391 (6)	
H27	0.3487	0.3318	0.3400	0.047*	
C28	0.4769 (2)	0.24996 (18)	0.30682 (12)	0.0421 (6)	
H28	0.5070	0.2973	0.2769	0.051*	
C29	0.5297 (2)	0.15893 (17)	0.31328 (11)	0.0347 (5)	
C30	0.48077 (17)	0.09257 (15)	0.35951 (9)	0.0253 (4)	
C31	0.6265 (2)	0.1311 (2)	0.27508 (11)	0.0434 (7)	
H31	0.6579	0.1756	0.2435	0.052*	
C32	0.6740 (2)	0.0432 (2)	0.28292 (11)	0.0418 (6)	
H32	0.7377	0.0265	0.2566	0.050*	
C33	0.62883 (17)	−0.02531 (18)	0.33100 (11)	0.0322 (5)	
C34	0.53289 (16)	−0.00081 (15)	0.36943 (10)	0.0239 (4)	
C35	0.67628 (18)	−0.11709 (18)	0.34117 (12)	0.0379 (6)	
H35	0.7416	−0.1353	0.3169	0.045*	
C36	0.62834 (18)	−0.18028 (17)	0.38602 (12)	0.0342 (5)	
H36	0.6598	−0.2427	0.3929	0.041*	
C37	0.53169 (17)	−0.15191 (14)	0.42193 (11)	0.0256 (4)	
C38	0.47268 (19)	−0.22145 (15)	0.46823 (12)	0.0326 (5)	
H38A	0.4480	−0.1877	0.5099	0.049*	
H38B	0.5250	−0.2734	0.4822	0.049*	
H38C	0.4065	−0.2487	0.4428	0.049*	

### Atomic displacement parameters (Å^2^)

**Table:**

	*U*^11^	*U*^22^	*U*^33^	*U*^12^	*U*^13^	*U*^23^
O1	0.0286 (8)	0.0431 (9)	0.0180 (7)	−0.0085 (6)	0.0034 (6)	0.0057 (6)
O2	0.0271 (8)	0.0398 (8)	0.0145 (6)	−0.0042 (6)	0.0010 (5)	−0.0001 (6)
O3	0.0188 (7)	0.0367 (8)	0.0272 (7)	0.0001 (6)	−0.0012 (6)	0.0025 (6)
O4	0.0218 (7)	0.0401 (8)	0.0208 (7)	−0.0060 (6)	0.0042 (5)	0.0038 (6)
O5	0.0260 (7)	0.0373 (8)	0.0204 (7)	−0.0079 (6)	0.0032 (5)	−0.0083 (6)
O6	0.0290 (7)	0.0263 (7)	0.0122 (6)	−0.0009 (5)	0.0024 (5)	0.0003 (5)
O7	0.0234 (7)	0.0229 (7)	0.0191 (6)	−0.0006 (5)	0.0035 (5)	−0.0034 (5)
O8	0.0170 (6)	0.0313 (7)	0.0193 (7)	−0.0010 (5)	0.0029 (5)	−0.0039 (5)
N1	0.0181 (8)	0.0210 (8)	0.0256 (8)	0.0010 (6)	0.0007 (6)	−0.0010 (6)
N2	0.0229 (8)	0.0232 (8)	0.0264 (8)	0.0045 (6)	−0.0002 (6)	−0.0027 (6)
N3	0.0280 (9)	0.0243 (9)	0.0229 (8)	0.0006 (7)	−0.0024 (7)	0.0001 (6)
N4	0.0174 (7)	0.0246 (8)	0.0215 (8)	−0.0004 (6)	−0.0010 (6)	−0.0052 (6)
C1	0.0231 (9)	0.0168 (8)	0.0152 (8)	0.0017 (7)	0.0010 (7)	0.0004 (6)
C2	0.0190 (9)	0.0181 (8)	0.0152 (8)	0.0010 (6)	0.0022 (6)	−0.0010 (6)
C3	0.0190 (9)	0.0212 (9)	0.0152 (8)	0.0024 (7)	0.0002 (6)	0.0006 (7)
C4	0.0229 (9)	0.0235 (9)	0.0155 (8)	0.0028 (7)	0.0027 (7)	0.0003 (7)
C5	0.0211 (9)	0.0223 (9)	0.0212 (9)	−0.0010 (7)	0.0027 (7)	−0.0035 (7)
C6	0.0208 (9)	0.0177 (8)	0.0133 (8)	−0.0016 (7)	0.0036 (6)	−0.0010 (6)
C7	0.0188 (8)	0.0166 (8)	0.0156 (8)	−0.0012 (6)	0.0026 (6)	0.0005 (6)
C8	0.0164 (8)	0.0204 (9)	0.0160 (8)	−0.0008 (6)	−0.0018 (6)	−0.0004 (6)
C9	0.0170 (8)	0.0252 (9)	0.0153 (8)	0.0024 (7)	0.0013 (6)	−0.0013 (7)
C10	0.0205 (9)	0.0172 (8)	0.0172 (8)	0.0000 (7)	0.0020 (7)	0.0016 (6)
C11	0.0310 (11)	0.0250 (10)	0.0300 (11)	0.0021 (8)	0.0003 (8)	0.0029 (8)
C12	0.0221 (9)	0.0220 (9)	0.0257 (10)	0.0010 (7)	−0.0033 (7)	−0.0006 (7)
C13	0.0245 (10)	0.0218 (10)	0.0318 (11)	0.0062 (7)	−0.0020 (8)	−0.0048 (8)
C14	0.0216 (9)	0.0276 (10)	0.0283 (10)	0.0037 (7)	0.0022 (8)	−0.0062 (8)
C15	0.0187 (9)	0.0249 (9)	0.0221 (9)	0.0004 (7)	−0.0027 (7)	−0.0041 (7)
C16	0.0173 (9)	0.0228 (9)	0.0213 (9)	−0.0015 (7)	−0.0029 (7)	−0.0016 (7)
C17	0.0205 (9)	0.0316 (11)	0.0214 (9)	−0.0034 (8)	−0.0002 (7)	−0.0025 (8)
C18	0.0268 (10)	0.0267 (10)	0.0222 (9)	−0.0051 (8)	−0.0023 (8)	0.0012 (7)
C19	0.0241 (9)	0.0206 (9)	0.0223 (9)	−0.0021 (7)	−0.0049 (7)	−0.0026 (7)
C20	0.0212 (9)	0.0207 (9)	0.0222 (9)	0.0010 (7)	−0.0036 (7)	−0.0032 (7)
C21	0.0315 (11)	0.0226 (10)	0.0303 (11)	−0.0020 (8)	−0.0085 (8)	0.0010 (8)
C22	0.0328 (11)	0.0211 (10)	0.0357 (11)	0.0050 (8)	−0.0072 (9)	−0.0024 (8)
C23	0.0249 (10)	0.0258 (10)	0.0302 (10)	0.0035 (8)	−0.0038 (8)	−0.0056 (8)
C24	0.0323 (12)	0.0307 (11)	0.0432 (13)	0.0112 (9)	0.0034 (10)	−0.0051 (9)
C25	0.0454 (14)	0.0277 (11)	0.0444 (13)	0.0117 (10)	−0.0056 (11)	−0.0029 (10)
C26	0.0396 (12)	0.0256 (10)	0.0268 (10)	−0.0002 (9)	−0.0113 (9)	−0.0008 (8)
C27	0.0559 (16)	0.0267 (11)	0.0334 (12)	−0.0054 (10)	−0.0178 (11)	0.0059 (9)
C28	0.0616 (17)	0.0373 (13)	0.0263 (11)	−0.0224 (12)	−0.0150 (11)	0.0104 (9)
C29	0.0425 (13)	0.0411 (13)	0.0202 (10)	−0.0199 (10)	−0.0035 (9)	0.0016 (9)
C30	0.0283 (10)	0.0304 (10)	0.0168 (9)	−0.0091 (8)	−0.0024 (7)	−0.0017 (7)
C31	0.0448 (14)	0.0651 (18)	0.0203 (10)	−0.0326 (13)	0.0019 (9)	−0.0003 (10)
C32	0.0334 (12)	0.0664 (18)	0.0265 (11)	−0.0236 (12)	0.0105 (9)	−0.0155 (11)
C33	0.0211 (10)	0.0505 (14)	0.0252 (10)	−0.0123 (9)	0.0036 (8)	−0.0151 (9)
C34	0.0183 (9)	0.0321 (11)	0.0215 (9)	−0.0074 (8)	0.0015 (7)	−0.0055 (8)
C35	0.0176 (10)	0.0558 (15)	0.0404 (13)	−0.0023 (9)	0.0035 (9)	−0.0252 (11)
C36	0.0228 (10)	0.0381 (12)	0.0412 (12)	0.0078 (9)	−0.0039 (9)	−0.0193 (10)
C37	0.0200 (9)	0.0268 (10)	0.0297 (10)	0.0030 (7)	−0.0044 (8)	−0.0090 (8)
C38	0.0321 (11)	0.0244 (10)	0.0408 (12)	0.0035 (8)	−0.0062 (9)	−0.0006 (9)

### Geometric parameters (Å, º)

**Table:**

O1—C4	1.275 (2)	C15—C16	1.402 (3)
O2—C4	1.244 (2)	C15—C17	1.431 (3)
O3—C5	1.226 (2)	C16—C20	1.432 (3)
O4—C5	1.295 (2)	C17—C18	1.356 (3)
O4—H1O	0.853 (10)	C17—H17	0.9500
O5—C9	1.211 (2)	C18—C19	1.430 (3)
O6—C9	1.320 (2)	C18—H18	0.9500
O6—H2O	0.850 (10)	C19—C21	1.412 (3)
O7—C10	1.223 (2)	C19—C20	1.415 (3)
O8—C10	1.309 (2)	C21—C22	1.363 (3)
O8—H3O	0.851 (10)	C21—H21	0.9500
N1—C12	1.333 (2)	C22—C23	1.420 (3)
N1—C16	1.366 (2)	C22—H22	0.9500
N1—H1N	0.886 (10)	C23—C24	1.503 (3)
N2—C23	1.328 (3)	C24—H24A	0.9800
N2—C20	1.355 (3)	C24—H24B	0.9800
N3—C26	1.329 (3)	C24—H24C	0.9800
N3—C30	1.359 (3)	C25—C26	1.505 (3)
N4—C37	1.330 (3)	C25—H25A	0.9800
N4—C34	1.364 (3)	C25—H25B	0.9800
C1—C3^i^	1.399 (3)	C25—H25C	0.9800
C1—C2	1.423 (2)	C26—C27	1.415 (3)
C1—C4	1.521 (2)	C27—C28	1.362 (4)
C2—C3	1.395 (2)	C27—H27	0.9500
C2—C5	1.531 (3)	C28—C29	1.413 (4)
C3—C1^i^	1.399 (3)	C28—H28	0.9500
C3—H3	0.9500	C29—C30	1.417 (3)
C6—C8^ii^	1.396 (3)	C29—C31	1.433 (4)
C6—C7	1.402 (2)	C30—C34	1.445 (3)
C6—C9	1.507 (2)	C31—C32	1.349 (4)
C7—C8	1.395 (2)	C31—H31	0.9500
C7—C10	1.503 (3)	C32—C33	1.442 (3)
C8—C6^ii^	1.396 (3)	C32—H32	0.9500
C8—H8	0.9500	C33—C35	1.403 (3)
C11—C12	1.492 (3)	C33—C34	1.415 (3)
C11—H11A	0.9800	C35—C36	1.366 (3)
C11—H11B	0.9800	C35—H35	0.9500
C11—H11C	0.9800	C36—C37	1.411 (3)
C12—C13	1.409 (3)	C36—H36	0.9500
C13—C14	1.371 (3)	C37—C38	1.501 (3)
C13—H13	0.9500	C38—H38A	0.9800
C14—C15	1.413 (3)	C38—H38B	0.9800
C14—H14	0.9500	C38—H38C	0.9800
			
C5—O4—H1O	112.6 (18)	C20—C19—C18	120.15 (17)
C9—O6—H2O	109.7 (17)	N2—C20—C19	124.58 (18)
C10—O8—H3O	103.9 (16)	N2—C20—C16	117.70 (18)
C12—N1—C16	123.66 (17)	C19—C20—C16	117.71 (18)
C12—N1—H1N	120.4 (15)	C22—C21—C19	119.6 (2)
C16—N1—H1N	115.9 (15)	C22—C21—H21	120.2
C23—N2—C20	117.74 (18)	C19—C21—H21	120.2
C26—N3—C30	118.95 (19)	C21—C22—C23	120.28 (19)
C37—N4—C34	119.62 (18)	C21—C22—H22	119.9
C3^i^—C1—C2	117.94 (16)	C23—C22—H22	119.9
C3^i^—C1—C4	114.11 (15)	N2—C23—C22	121.8 (2)
C2—C1—C4	127.95 (17)	N2—C23—C24	116.98 (19)
C3—C2—C1	117.58 (17)	C22—C23—C24	121.24 (19)
C3—C2—C5	114.00 (15)	C23—C24—H24A	109.5
C1—C2—C5	128.39 (16)	C23—C24—H24B	109.5
C2—C3—C1^i^	124.48 (16)	H24A—C24—H24B	109.5
C2—C3—H3	117.8	C23—C24—H24C	109.5
C1^i^—C3—H3	117.8	H24A—C24—H24C	109.5
O2—C4—O1	122.35 (17)	H24B—C24—H24C	109.5
O2—C4—C1	117.46 (17)	C26—C25—H25A	109.5
O1—C4—C1	120.17 (16)	C26—C25—H25B	109.5
O3—C5—O4	121.32 (18)	H25A—C25—H25B	109.5
O3—C5—C2	119.44 (17)	C26—C25—H25C	109.5
O4—C5—C2	119.17 (16)	H25A—C25—H25C	109.5
C8^ii^—C6—C7	119.87 (16)	H25B—C25—H25C	109.5
C8^ii^—C6—C9	116.60 (15)	N3—C26—C27	121.9 (2)
C7—C6—C9	123.48 (16)	N3—C26—C25	116.8 (2)
C8—C7—C6	119.28 (17)	C27—C26—C25	121.4 (2)
C8—C7—C10	120.16 (15)	C28—C27—C26	119.5 (2)
C6—C7—C10	120.47 (16)	C28—C27—H27	120.3
C7—C8—C6^ii^	120.85 (16)	C26—C27—H27	120.3
C7—C8—H8	119.6	C27—C28—C29	120.2 (2)
C6^ii^—C8—H8	119.6	C27—C28—H28	119.9
O5—C9—O6	125.37 (17)	C29—C28—H28	119.9
O5—C9—C6	122.40 (17)	C28—C29—C30	116.7 (2)
O6—C9—C6	112.04 (15)	C28—C29—C31	123.6 (2)
O7—C10—O8	125.23 (17)	C30—C29—C31	119.7 (2)
O7—C10—C7	120.91 (16)	N3—C30—C29	122.7 (2)
O8—C10—C7	113.84 (15)	N3—C30—C34	118.23 (18)
C12—C11—H11A	109.5	C29—C30—C34	119.0 (2)
C12—C11—H11B	109.5	C32—C31—C29	121.5 (2)
H11A—C11—H11B	109.5	C32—C31—H31	119.2
C12—C11—H11C	109.5	C29—C31—H31	119.2
H11A—C11—H11C	109.5	C31—C32—C33	120.4 (2)
H11B—C11—H11C	109.5	C31—C32—H32	119.8
N1—C12—C13	118.27 (18)	C33—C32—H32	119.8
N1—C12—C11	118.29 (18)	C35—C33—C34	118.0 (2)
C13—C12—C11	123.41 (18)	C35—C33—C32	122.3 (2)
C14—C13—C12	120.16 (18)	C34—C33—C32	119.8 (2)
C14—C13—H13	119.9	N4—C34—C33	121.3 (2)
C12—C13—H13	119.9	N4—C34—C30	119.23 (18)
C13—C14—C15	120.83 (18)	C33—C34—C30	119.49 (19)
C13—C14—H14	119.6	C36—C35—C33	119.9 (2)
C15—C14—H14	119.6	C36—C35—H35	120.0
C16—C15—C14	117.30 (18)	C33—C35—H35	120.0
C16—C15—C17	118.90 (18)	C35—C36—C37	119.3 (2)
C14—C15—C17	123.76 (19)	C35—C36—H36	120.4
N1—C16—C15	119.75 (17)	C37—C36—H36	120.4
N1—C16—C20	118.73 (18)	N4—C37—C36	121.9 (2)
C15—C16—C20	121.51 (18)	N4—C37—C38	117.36 (18)
C18—C17—C15	120.61 (19)	C36—C37—C38	120.71 (19)
C18—C17—H17	119.7	C37—C38—H38A	109.5
C15—C17—H17	119.7	C37—C38—H38B	109.5
C17—C18—C19	121.10 (19)	H38A—C38—H38B	109.5
C17—C18—H18	119.4	C37—C38—H38C	109.5
C19—C18—H18	119.4	H38A—C38—H38C	109.5
C21—C19—C20	115.99 (19)	H38B—C38—H38C	109.5
C21—C19—C18	123.85 (19)		
			
C3^i^—C1—C2—C3	−0.5 (3)	C18—C19—C20—N2	177.97 (17)
C4—C1—C2—C3	−179.62 (17)	C21—C19—C20—C16	−179.72 (16)
C3^i^—C1—C2—C5	−178.40 (17)	C18—C19—C20—C16	−0.6 (3)
C4—C1—C2—C5	2.4 (3)	N1—C16—C20—N2	−0.6 (2)
C1—C2—C3—C1^i^	0.5 (3)	C15—C16—C20—N2	−179.19 (16)
C5—C2—C3—C1^i^	178.73 (17)	N1—C16—C20—C19	178.12 (15)
C3^i^—C1—C4—O2	−10.8 (2)	C15—C16—C20—C19	−0.5 (3)
C2—C1—C4—O2	168.41 (18)	C20—C19—C21—C22	0.4 (3)
C3^i^—C1—C4—O1	167.52 (17)	C18—C19—C21—C22	−178.69 (18)
C2—C1—C4—O1	−13.3 (3)	C19—C21—C22—C23	1.0 (3)
C3—C2—C5—O3	15.2 (3)	C20—N2—C23—C22	1.1 (3)
C1—C2—C5—O3	−166.75 (18)	C20—N2—C23—C24	−178.25 (17)
C3—C2—C5—O4	−161.82 (17)	C21—C22—C23—N2	−1.8 (3)
C1—C2—C5—O4	16.2 (3)	C21—C22—C23—C24	177.49 (19)
C8^ii^—C6—C7—C8	0.0 (3)	C30—N3—C26—C27	−1.1 (3)
C9—C6—C7—C8	−177.05 (16)	C30—N3—C26—C25	179.39 (17)
C8^ii^—C6—C7—C10	−176.53 (16)	N3—C26—C27—C28	2.7 (3)
C9—C6—C7—C10	6.4 (3)	C25—C26—C27—C28	−177.8 (2)
C6—C7—C8—C6^ii^	0.0 (3)	C26—C27—C28—C29	−1.7 (3)
C10—C7—C8—C6^ii^	176.54 (16)	C27—C28—C29—C30	−0.8 (3)
C8^ii^—C6—C9—O5	64.4 (2)	C27—C28—C29—C31	178.0 (2)
C7—C6—C9—O5	−118.4 (2)	C26—N3—C30—C29	−1.5 (3)
C8^ii^—C6—C9—O6	−110.89 (18)	C26—N3—C30—C34	179.41 (16)
C7—C6—C9—O6	66.3 (2)	C28—C29—C30—N3	2.5 (3)
C8—C7—C10—O7	−158.38 (17)	C31—C29—C30—N3	−176.38 (18)
C6—C7—C10—O7	18.2 (3)	C28—C29—C30—C34	−178.51 (17)
C8—C7—C10—O8	20.0 (2)	C31—C29—C30—C34	2.7 (3)
C6—C7—C10—O8	−163.44 (16)	C28—C29—C31—C32	−180.0 (2)
C16—N1—C12—C13	−2.0 (3)	C30—C29—C31—C32	−1.2 (3)
C16—N1—C12—C11	176.50 (16)	C29—C31—C32—C33	−0.6 (3)
N1—C12—C13—C14	1.8 (3)	C31—C32—C33—C35	−179.5 (2)
C11—C12—C13—C14	−176.64 (18)	C31—C32—C33—C34	1.0 (3)
C12—C13—C14—C15	−0.4 (3)	C37—N4—C34—C33	−1.0 (3)
C13—C14—C15—C16	−0.8 (3)	C37—N4—C34—C30	177.42 (16)
C13—C14—C15—C17	176.99 (17)	C35—C33—C34—N4	−0.6 (3)
C12—N1—C16—C15	0.8 (3)	C32—C33—C34—N4	178.94 (17)
C12—N1—C16—C20	−177.88 (16)	C35—C33—C34—C30	−179.09 (17)
C14—C15—C16—N1	0.7 (3)	C32—C33—C34—C30	0.5 (3)
C17—C15—C16—N1	−177.25 (16)	N3—C30—C34—N4	−1.7 (3)
C14—C15—C16—C20	179.30 (16)	C29—C30—C34—N4	179.23 (16)
C17—C15—C16—C20	1.4 (3)	N3—C30—C34—C33	176.79 (16)
C16—C15—C17—C18	−1.1 (3)	C29—C30—C34—C33	−2.3 (3)
C14—C15—C17—C18	−178.89 (18)	C34—C33—C35—C36	1.3 (3)
C15—C17—C18—C19	0.0 (3)	C32—C33—C35—C36	−178.27 (19)
C17—C18—C19—C21	179.92 (18)	C33—C35—C36—C37	−0.4 (3)
C17—C18—C19—C20	0.9 (3)	C34—N4—C37—C36	2.0 (3)
C23—N2—C20—C19	0.4 (3)	C34—N4—C37—C38	−175.67 (16)
C23—N2—C20—C16	179.00 (16)	C35—C36—C37—N4	−1.4 (3)
C21—C19—C20—N2	−1.1 (3)	C35—C36—C37—C38	176.27 (18)

Symmetry codes: (i) −*x*+1, −*y*+1, −*z*+1; (ii) −*x*+2, −*y*, −*z*+1.

### Hydrogen-bond geometry (Å, º)

**Table:**

*D*—H···*A*	*D*—H	H···*A*	*D*···*A*	*D*—H···*A*
O4—H1o···O1	0.85 (2)	1.55 (2)	2.403 (2)	176 (3)
O6—H2o···O2^iii^	0.85 (1)	1.74 (1)	2.577 (2)	168 (2)
O8—H3o···N4^iv^	0.85 (2)	1.79 (2)	2.636 (2)	173 (2)
N1—H1n···O3^v^	0.89 (2)	2.41 (2)	3.257 (2)	161 (2)
N1—H1n···O4^v^	0.89 (2)	2.35 (2)	2.957 (2)	126 (2)
C13—H13···O7^vi^	0.95	2.28	3.225 (3)	171
C28—H28···O5^vii^	0.95	2.40	3.320 (3)	162

Symmetry codes: (iii) *x*+1/2, −*y*+1/2, *z*+1/2; (iv) −*x*+1, −*y*, −*z*+1; (v) −*x*+3/2, *y*+1/2, −*z*+1/2; (vi) *x*−1/2, −*y*+3/2, *z*−1/2; (vii) *x*−1/2, −*y*+1/2, *z*−1/2.
